# Evaluation of Immunogenic Effect of *Toxoplasma gondii* Recombinant SAG-1 Antigen with Propranolol as Adjuvant in BALB/c Mice

**DOI:** 10.15171/apb.2019.073

**Published:** 2019-10-24

**Authors:** Esmaeil Abasi, Shahram Shahabi, Majid Golkar, Peyman Khezri, Habib Mohammadzadeh Hajipirloo

**Affiliations:** ^1^Department of Parasitology and Mycology, Faculty of Medicine, Urmia University of Medical Sciences, Urmia, Iran.; ^2^Department of Immunology, Faculty of Medicine, Urmia University of Medical Sciences, Urmia, Iran.; ^3^Molecular Parasitology Laboratory, Department of Parasitology, Pasteur Institute of Iran, Tehran, Iran.

**Keywords:** Adjuvant, Propranolol, r SAG-1, *Toxoplasma gondii*, Vaccine

## Abstract

***Purpose:*** Propranolol as a novel adjuvant, was used to evaluate the immunogenic effect of three doses of recombinant SAG-1 (rSAG-1) antigen of *Toxoplasma gondii* in BALB/c mice for finding the optimal dose, and was compared with efficacy of tachyzoite lysate antigen (TLA).

*** Methods:*** Eight different groups of 15 BALB/c mice received different volumes of the immunogenic material (three doses of r SAG-1 and one dose of TLA antigens), with or without propranolol adjuvant, subcutaneously. The control group mice received only PBS. Three weeks after the last immunization, the serum levels of IgG2a, IgG1 and IgG total antibodies against TLA, splenic interleukin-5 (IL-5) and Interferon-gamma (IFN-γ) (produced against TLA) and the splenic lymphocyte proliferation after adding TLA were measured to evaluate humoral and cellular immune responses. Challenge test was performed by subcutaneously injection of 1000 alive and active tachyzoites in to five mice per each group and survival days for each group of mice were recorded.

***Results:*** The mice group that received propranolol adjuvant and 20 µg of r SAG-1 antigen per dose of injection showed significantly more IFN-γ production, more proliferation of splenic lymphocytes and higher anti-TLA-specific IgG2a production (three main indexes for cell mediated immunity) in comparison with other groups. Moreover, in the challenge test, this group of mice had a significantly increased survival time, indicating the positive effect of propranolol in the more stimulating of cellular immunity that is necessary for toxoplasmosis prevention or suppress.

*** Conclusion:*** Our results showed that *T. gondii* rSAG-1 antigen in combination with propranolol as adjuvant (which can induce Th1 related responses) are good candidates for further study to a vaccine design.

## Introduction

The protozoan parasite *Toxoplasma gondii* belongs to Apicomplexa phylum and has a worldwide distribution.^1^ The parasite has a wide host range and is able to infect almost all warm-blooded animals as intermediate hosts.^2-4^ On the basis of serological studies, it has been estimated that One-third of the adult population in several countries are infected with this parasite.^5^ The prevalence of toxoplasmosis with clinical symptoms is much lower than its infection rate.^6,7^ It is also very important in the veterinary field as it causes abortion and stillbirth in domestic animals. In addition, domestic animal infection can be considered as a routine way for human infection through meat consumption (containing tissue cysts).^8,9^ Sexual replication occurs in the small intestine cells of the definitive hosts (Felidae family), while asexual replication occurs in the nucleated cells of almost all warm-blooded animals and humans.^7^ If the infection occurs for the first time during pregnancy, it can lead to abortion or a fetus birth with physical or mental abnormalities.^10^ Infection in people with a deficient immune system can lead to eye and brain involvement and in some cases can lead to disseminated and fatal infection.^11^ Some studies support the contamination with *T. gondii* as a risk factor for schizophrenia^12^ or independently confirm that personality profile is affected by latent toxoplasmosis.^1^ So, medical and veterinary importance of this parasite is the primary reason of scientists’ efforts for developing an effective vaccine against toxoplasmosis.^13^ The aim of vaccination is the production of strong long-lasting immune-protecting responses and adjuvants can be used to enhance stimulation of the immune system.^14^ Both humoral immunity and cellular immunity are essential for the control of toxoplasmosis, although cell-mediated immunity plays a more important role.^15^ Recently, subunit vaccines are being widely used.^9^ These vaccines have fewer side effects in comparison with those that use attenuated or weakened live organisms, but they are poorly recognized and presented by antigen-presenting cells. Therefore, not only they need to be optimized, but they should also be used along with adjuvants.^16^ As *T. gondii* is an obligatory intracellular protozoa, so, the cell mediated immunity is necessary for infection control,^17^ and designing a protective vaccine model would require an adjuvant that be able to lead the immune reactions to Th1 and cellular immunity. Propranolol is a nonselective beta-adrenoceptor antagonist,^18^ and it was used as an adjuvant in this study (based on our previous experience on propranolol and its beneficial effects as adjuvant^19^) in association with tachyzoite surface antigen (SAG-1) as an antigen. Use of propranolol as an adjuvant will suppress the inhibitory effects of the sympathetic nervous system on the production of cytokines related to cellular immunity, such as Interleukin 12 (IL-12), Tumor necrosis factor-α (TNFα), andinterferon-gamma (IFN-γ, and therefore the immune responses will shift to Th1 that are necessary to control the disease.^20,21^ The purpose of the present study was based on the evaluation of the immunogenic influences of three different doses (5, 10, and 20 µg per injection) of r SAG-1 antigen in association with or without propranolol as adjuvant and to compare the immunogenicity potential of r SAG-1 and tachyzoite lysate antigen (TLA) (at 20 µg per injection concentration) to lead the immune responses to Th1 profile and ultimately, probable protection the mice against the pathogenicity of RH strain of *T. gondii*.

## Materials and Methods

### Mice

Inbred female BALB/c mice (6 to 8 week-old) were bought from Razi Institute of Iran and were used throughout the immunization experiments. The experimental stages were performed according to the prescribed rules of institutional animal use and care of the Urmia University of Medical Sciences, Urmia, Iran.

### Toxoplasma gondii proliferation

TLA was prepared according to a previously described method,^22^ and tachyzoites (RH strain) were proliferated and also cryopreserved for future studies via BALB/c mice intraperitoneal infection. The parasites in the aspirated fluid were used to produce antigens and for injection into other mice.

### Preparation of Toxoplasma lysate antigen

Aspirated fluid from mice peritonea was squeezed four to five times through a 30-guage needle and washed three times by cold centrifugation at 750 g. Then, the fluid was subjected to freeze-and-thaw cycles at −20°C and 4°C for three times. PMSF, EDTA, penicillin, and streptomycin were added to the sedimented parasites and ice sonication was performed for 15 times. Cold centrifuge at 750 *g* was performed on the sonicated suspension for 30 minutes. Using polyethylene glycol powder, dialyzing was performed to concentrate the supernatant and eliminate the anti-proteases from it. A 0.22-µ filter was used to sterilize the dialyzed fluid and then, its protein level was measured by Biuret method and finally transferred to −20°C until use in mice immunization process.

### Preparation of recombinant SAG-1 antigen

In this study, the SAG-1 antigen (amino acids 49 to 311, accession number: X14080.1) that obtained from the RH strain of *T. gondii*, was used for immunization process. Recombinant SAG-1 was expressed in Escherichia *coli* cells as inclusion bodies (IBs). The IBs were separated from the cell extract by centrifugation, and washed using IB washing buffers, as previously described,^23^ to remove bacterial impurities and cell debris. Subsequently, the IBs were solubilized using Tris-HCl buffer containing 8 M Urea, and rSAG-1 was purified by a single immobilized metal ion chromatography (IMAC – Bio-Rad Laboratories, Inc, USA). Purified protein was refolded by dialysis method in presence of reduced/oxidized glutathione, then analyzed by sodium dodecyl sulfate-polyacrylamide gel electrophoresis (SDS-PAGE), followed by dialyzing against phosphate-buffered saline (PBS), and finally preserved in aliquots at -70°C.

### Immunization

For the study, 135 BALB/c mice (described above) were randomly divided into nine groups and from15 animals per group, 5 were used for bleeding, 5 for the challenge experiment and 5 were kept as reserves to be used at different stages, if necessary, and then specific doses of the prepared solutions (listed in the Table 1) were subcutaneously injected to each mice group on the 1st, 14th, and 28th days.

**Table 1:** BALB/c mice groups subcutaneous injection protocols and doses

**Group name**	**Injections**			
	**rSAG-1 volume (µL)**	**TLA volume (µL)**	**Propranolol volume (µL)**	**PBS volume (µL)**
SAG1-5	50	-	-	100
SAG1-5PRO	50	-	50	50
SAG1-10	50	-	-	100
SAG1-10PRO	50	-	50	50
SAG1-20	50	-	-	100
SAG1-20PRO	50	-	50	50
TLA	-	50	-	100
TLA-PRO	­-	50	50	50
Control	-	-	-	150

rSAG-1: recombinant surface antigen 1; TLA: tachyzoite lysate antigen; PBS: phosphate-buffered saline; SAG1-5: 5 µg per dose of injection of r SAG-1 antigen. SAG1-5Pro: 5 µg per dose of injection of r SAG-1 antigen in combination with propranolol adjuvant. SAG1-10: 10 µg per dose of injection of r SAG-1 antigen. SAG1-10PRO: 10 µg per dose of injection of r SAG-1 antigen in combination with propranolol adjuvant. SAG1-20: 20 µg per dose of injection of r SAG-1 antigen. SAG1-20PRO: 20 µg per dose of injection of r SAG-1 antigen in combination with propranolol adjuvant. TLA: 20 µg per dose of injection of TLA antigen. TLA-PRO: 20 µg per dose of injection of TLA antigen in combination with propranolol adjuvant.

### Splenic cells proliferation assay

After 3 weeks of the last immunization, five mice from each group selected to be used in splenic lymphocyte proliferation test. For mice anesthetizing, an intraperitoneal injection of 50 µL of a mixture including two parts of ketamine (66.4 mg/kg), one part of xylazine (6.64 mg/kg), and three parts of PBS, was used. After anesthetizing, cardiac puncture method was used to mice bleeding and serum sample of each mouse was transferred to −20°C until use for detecting of specific antibodies levels against TLA antigen. Then, under sterile conditions, the spleen of each mouse was removed and was homogenized using a glass homogenizer in RPMI 1640 primary culture medium (PCM). After centrifuging the resulted suspension at 350 g, 2 mL of PCM and 13 mL of 0.9% ammonium chloride were added to the sediment until the RBCs be lysed. Next to keeping the solution for 5 min at room temperature and centrifugation at 350 g, the splenocytes were washed twice with PCM. The sediment was dissolved in 2 mL of sterile RPMI 1640 complete culture medium (CCM), and the cell count and viability determination were performed using trypan blue dye. The cells were added in 96-well culture microplates (in duplicate) at a concentration of 10^5^ cells/well in 100 µL RPMI 1640 CCM. Then, the stimulation of lymphocytes of antigen wells (those that received stimulative antigen) was performed by adding 15 µL of TLA antigen, followed by adjusting the volumes to 100 µL per well. In the control wells, 100 µL of RPMI medium was added. Incubation at 37°C with 5% CO2 was performed for two days. Then, 20 µL of MTT solution (3-(4,5-dimethylthiazol-2-yl)-2,5-diphenyltetrazolium bromide) was added to each well (5 mg/mL). The microplates were incubated again (for 4 h) under the similar conditions and then centrifuged at 350 g for 10 min. The supernatant was discarded from all wells, and followed by adding 100 µL of DMSO solution to all wells for solubilizing the resulting crystals. Measuring of optical density (OD) for each well was performed by an ELISA reader (model Awareness Technologies Stat Fax 2100 Palm City, FL, USA) at 540 nm.^24^ The results of lymphocyte proliferation was calculated and demonstrated as the stimulation index (SI) using following formula.^25^

$$SI=25\frac{\text{Mean OD of stimulated cells}-\text{Mean OD of blank}}{\text{Mean OD of unstimulated cells}}$$

### Assay of cytokines

The splenocytes were cultured similar to the method illustrated for the lymphocyte proliferation assay but the incubation time was 72 h. The microplates were centrifuged at 350 g for 10 min and the aspirated supernatant of each well, was transferred to −80°C until use for measuring IFN-γ and interleukin-5 (IL-5) levels^26^ using a commercial ELISA kit (Mouse IFN-γ ELISA development kit, MABTECH, Product code: 3321-1H-20, and Mouse IL-5 ELISA development kit, MABTECH, Product code: 3391-1H-6).^27^

### Assay for antibodies

The production of different IgG isotypes is relative to T-cell-specific cytokines. IgG1 is a Th2-specific isotype and is produced via the direct influence of IL-4 on B lymphocytes (IL-4 is the primary cytokine and characteristic of Th2).^28^ IgG2a belongs to the Th1-specific subclass, and its production is dependent on the direct influence of IFN-γ on B lymphocytes.^29^ Therefore, serum TLA-specific IgG total, IgG1, and IgG2a levels were analyzed to evaluate Th1 and or Th2 responses among the various experimental mice groups. Mice serum samples that were isolated in the lymphocyte proliferation step were diluted, and TLA-specific IgG total, IgG1, and IgG2a levels were determined using the ELISA method described by Voller et al^30^ with some modifications, as described below.

A concentration of 10 µg/mL in 0.05 M carbonate–bicarbonate buffer of TLA was prepared for Coating step (by adding 100 µL of TLA into 96-well microplates), followed by 12–24 h of incubation at 4°C–8°C. After washing with PBS-Tween, 1% BSA in PBS solution was used for blocking step. After a repeated washing, 100 µL serum samples (1:100 diluted in PBS/BSA/Tween 20) were added to each well (in duplicate) and followed by 2 h incubation at room temperature. After another wash, 100 µL of goat anti-mouse IgG2a or IgG1 (1:2000 dilution) and IgG total (1/4000 dilution) was dispensed in to all related wells and followed by 2 h incubation and another wash step. At next step, 100 µL of 3,3ʹ,5,5ʹ-tetramethylbenzidine (TMB) was dispensed in to all wells (TMB was prepared and used at the same time). H2SO4 was used to stop color developing in wells after incubation. The optical densities of each well was measured by the ELISA reader (model Awareness Technologies Stat Fax 2100 Palm City, FL, USA), and the increase in antibody levels were determined by calculating and comparing the differences between obtained values for various groups.^30^

### Challenge of immunized mice

The challenge test was performed with subcutaneous injection of 1000 alive and active RH strain tachyzoites to five mice from each group on the 20th day after the last immunization and the survival days of each group of mice were monitored until 3 weeks.^31,32^

### Statistical analysis

For comparison of the MTT test results and the mean values of cytokines and antibody levels, ANOVA was used. The survival test was performed by the Kaplan–Meier analysis. SPSS software was used for the statistical analyses. The statistical significance differences are expressed as mean using GraphPad Prism version 6 (GraphPad Software, San Diego, CA). *P* value of <0.05 was considered as a statistically significant difference.

### Theory/calculation

Each individual antigen induces a specific type of immune response and so, identification of more immunogenic antigens for each parasite can be too helpful in designing the preventive strategies of the disease such as vaccination methods. In addition, the use of appropriate adjuvants which can shift the immune system to the desired type (cellular or humoral immunity) can bring better results. Thus, in this study, with approach to stimulate cell mediated immunity, we applied r SAG-1 and TLA antigen and compared their ability in activation of cellular immune system with and without propranolol as an adjuvant (with a capacity to stimulate the cell mediated immunity).

## Results and Discussion

### Lymphocyte proliferation assay

**Figure 1:**
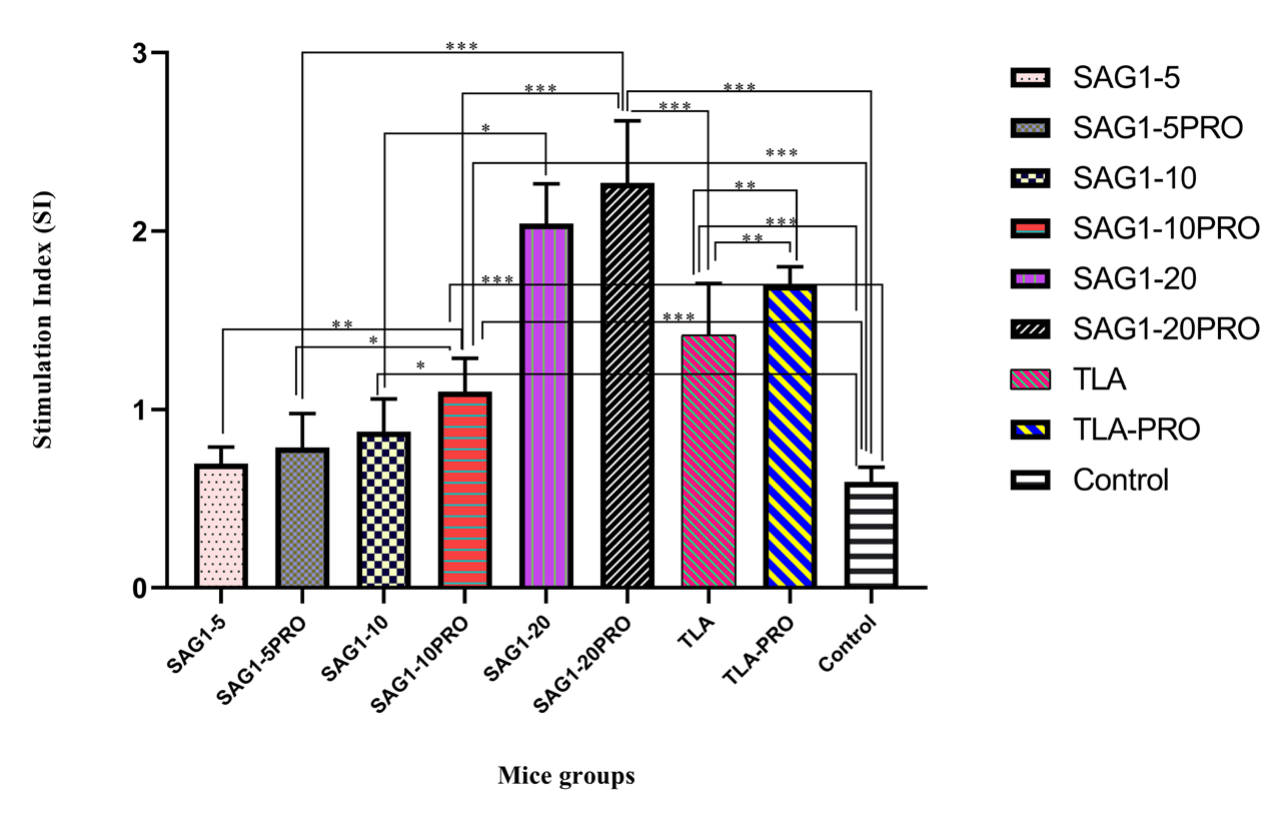
**Effect of administering rSAG-1, TLA and propranolol adjuvant on splenic lymphocytes proliferation. **SAG1-5: 5 µg per dose of injection of rSAG-1 antigen, SAG1-5PRO: 5 µg per dose of injection of rSAG-1 antigen in combination with propranolol adjuvant, SAG1-10: 10 µg per dose of injection of rSAG-1 antigen, SAG1-10PRO: 10 µg per dose of injection of rSAG-1 antigen in combination with propranolol adjuvant, SAG1-20: 20 µg per dose of injection of r SAG-1 antigen, SAG1-20PRO: 20 µg per dose of injection of rSAG-1 antigen in combination with propranolol adjuvant, TLA: 20 µg per dose of injection of TLA antigen, TLA-PRO: 20 µg per dose of injection of TLA antigen in combination with propranolol adjuvant, control: 150 µg per dose of injection of PBS. *P value < 0.05; **P value < 0.01; ***P value < 0.001

Splenic lymphocyte proliferation responses are known as one of the indicators of cellular immunity assessment. The SI, achieved from the mice splenocyte stimulation by the TLA antigen are presented in Figure 1. According to the MTT test results, the lymphocyte proliferation response was significantly higher among the groups that received both antigen and adjuvant (propranolol) than that in the groups that did not receive the adjuvant. The increase in lymphocyte proliferation response in the group that received r SAG-1 at a concentration of 20 µg per dose of injection + propranolol is several times higher than that in the groups that received lower doses of r SAG-1 and the control group and further higher than that in the TLA groups, implying that the difference between SAG1-20Pro group and all other groups was statistically significant (*P* < 0.001).

### IFN-γ production

**Figure 2:**
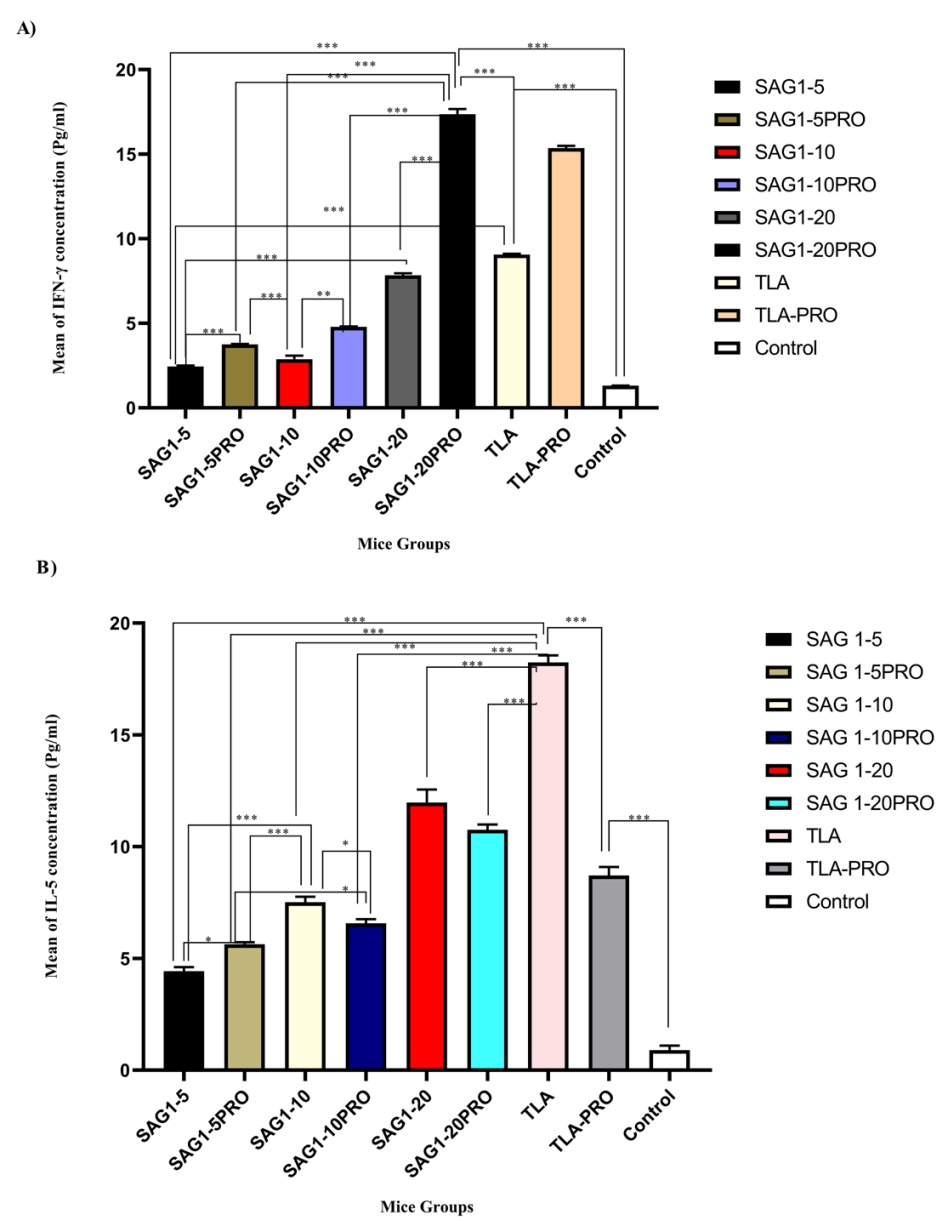
**Effect of administering rSAG-1, TLA and propranolol adjuvant on the splenic IFN-γ(A) and IL-5 (B) production**. SAG1-5: 5 µg per dose of injection of rSAG-1 antigen, SAG1-5PRO: 5 µg per dose of injection of rSAG-1 antigen in combination with propranolol adjuvant, SAG1-10: 10 µg per dose of injection of rSAG-1 antigen, SAG1-10PRO: 10 µg per dose of injection of r SAG-1 antigen in combination with propranolol adjuvant, SAG1-20: 20 µg per dose of injection of rSAG-1 antigen, SAG1-20PRO: 20 µg per dose of injection of rSAG-1 antigen in combination with propranolol adjuvant, TLA: 20 µg per dose of injection of TLA antigen , TLA-PRO: 20 µg per dose of injection of TLA antigen in combination with propranolol adjuvant, control: 150 µg per dose of injection of PBS. *P value < 0.05; **P value < 0.01; ***P value < 0.001.

IFN-γ is the major cytokine in the cellular responses against *T. gondii* infections. The IFN-γ assay results are shown in Figure 2A, which shows that the splenic IFN-γ levels are higher in the groups that received propranolol compared to that in the adjuvant-free groups. The increase in IFN-γ levels in the group that received the recombinant SAG-1 at a concentration of 20 µg per dose of injection + propranolol is several times higher than that in the groups that received lower doses of r SAG-1 and the control groups and further higher than that in the TLA group. Therefore, the difference in SAG1-20 + PRO group in comparison with all other test groups was statistically significant (*P* < 0.001). Moreover, the group that received 20 µg per dose of injection of TLA with propranolol showed a statistically significant increased splenic IFN-γ production in comparison with other test and also control groups (*P* < 0.001).

### Assaying of IL-5

The IL-5 levels (produced by splenocytes) were determined for the evaluation of Th2 responses among the various experimental groups, and the results are shown in Figure2B. As shown in the figure, the splenic IL-5 levels are higher in the groups that did not receive propranolol compared to those in the groups that received propranolol. The increase in splenic IL-5 levels in the groups that received TLA at a concentration of 20 µg per dose of injection without propranolol is higher than that in all eight other groups in this study (*P* < 0.001). Moreover, the group that received r SAG-1 at 20 µg per dose of injection showed significantly increased splenic IL-5 levels compared to those in the groups that received lower doses of r SAG-1.

### Assay of IgG subclasses

#### IgG total

**Figure 3:**
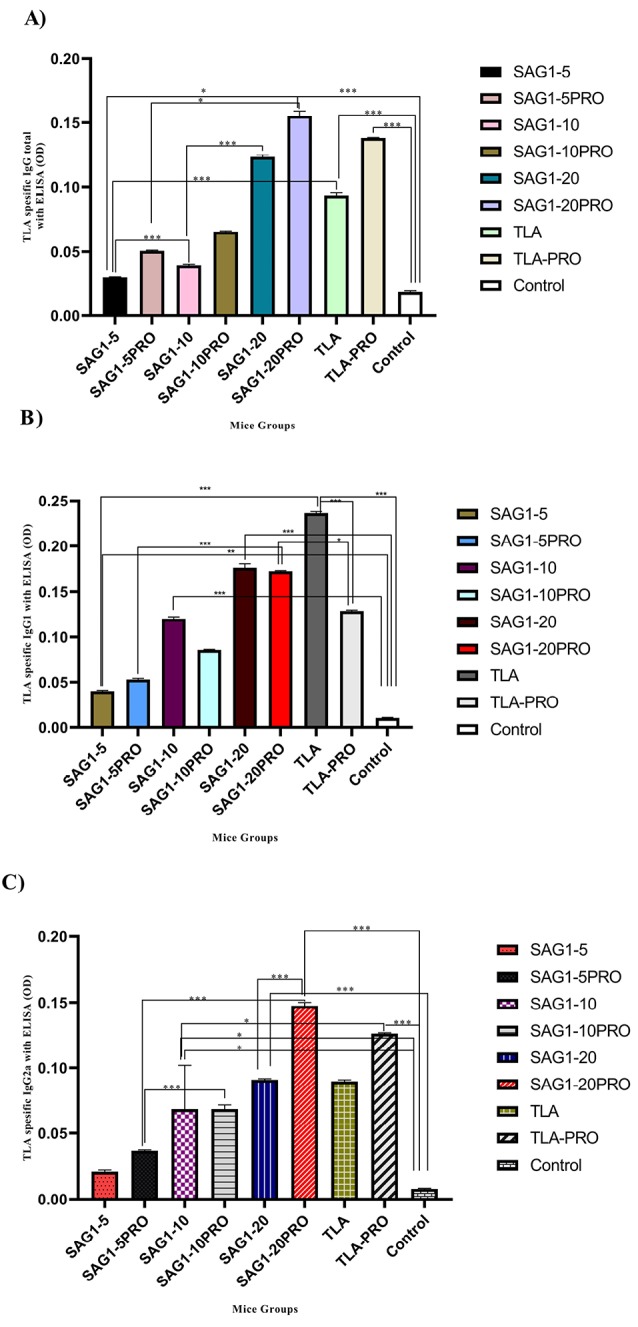
**Effect of administering rSAG-1, TLA and propranolol adjuvant on the mice serum TLA-specific IgG total (A), IgG1 (B) and IgG2a (C) production.** SAG1-5: 5 µg per dose of injection of rSAG-1 antigen, SAG1-5PRO: 5 µg per dose of injection of rSAG-1 antigen in combination with propranolol adjuvant, SAG1-10: 10 µg per dose of injection of rSAG-1 antigen, SAG1-10PRO: 10 µg per dose of injection of rSAG-1 antigen in combination with propranolol adjuvant, SAG1-20: 20 µg per dose of injection of rSAG-1 antigen, SAG1-20PRO: 20 µg per dose of injection of rSAG-1 antigen in combination with propranolol adjuvant, TLA: 20 µg per dose of injection of TLA antigen, TLA-PRO: 20 µg per dose of injection of TLA antigen in combination with propranolol adjuvant, control: 150 µg per dose of injection of PBS. *P value < 0.05; **P value < 0.01; ***P value < 0.001.

Anti-TLA-specific IgG total assay shows that the levels of anti-TLA-specific IgG total are higher in the groups that received propranolol compared to those in the groups that did not receive propranolol (Figure 3A). The increase in the anti-TLA-specific IgG total levels in the group that received the recombinant SAG-1 at a concentration of 20 µg per dose of injection + propranolol is higher than that in the other groups that received TLA, lower doses of r SAG-1, and in the control group. Thus, the SAG1-20 + PRO group showed a highly statistically significant discrepancy in comparison with all other groups (*P* < 0.001). In addition, increased splenic IgG total production observed in the groups that were injected by 20 µg per dose of injection of TLA with propranolol and 20 µg per dose of injection of rSAG-1 without propranolol and showed a statistically significance compared to all other groups (*P* < 0.001).

#### Assaying of IgG1

The serum levels of IgG2a (specific for TLA) are higher in the groups that did not receive propranolol compared to those in the groups that received propranolol (Figure 3B). The increase in the anti-TLA-specific IgG1 levels in the group that received TLA at a concentration of 20 µg per dose of injection, without propranolol, is higher than that in all the other study groups (*P* < 0.001).

#### Assaying of IgG2a

Increased serum levels of IgG2a antibody (produced against TLA) are seen among the groups that received propranolol compared to those in the groups that did not receive propranolol (Figure 3C). The increase in the anti-TLA-specific IgG2a levels in the group that received the recombinant SAG-1 at a concentration of 20 µg per dose of injection + propranolol is higher than that in the groups that received TLA, lower doses of r SAG-1, and in the control group. Therefore, the difference between SAG1-20 + PRO group and all other study groups was statistically significant (*P* < 0.001). Furthermore, a similar situation was observed in group that was injected by 20 µg of TLA with propranolol (*P* < 0.001).

#### Challenge and survival test

**Figure 4:**
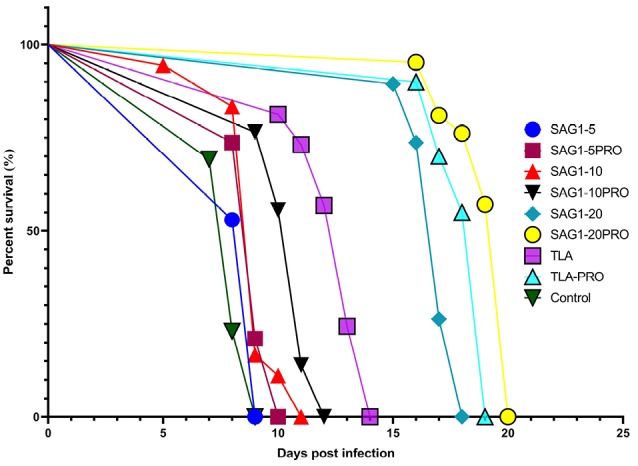
**Survival days number for five mice from each group after challenge with lethal dose of *****T. gondii*** **RH strain.** SAG1-5: 5 µg per dose of injection of rSAG-1 antigen, SAG1-5PRO: 5 µg per dose of injection of rSAG-1 antigen in combination with propranolol adjuvant, SAG1-10: 10 µg per dose of injection of rSAG-1 antigen, SAG1-10PRO: 10 µg per dose of injection of rSAG-1 antigen in combination with propranolol adjuvant, SAG1-20: 20 µg per dose of injection of rSAG-1 antigen, SAG1-20PRO: 20 µg per dose of injection of rSAG-1 antigen in combination with propranolol adjuvant, TLA: 20 µg per dose of injection of TLA antigen, TLA-PRO: 20 µg per dose of injection of TLA antigen in combination with propranolol adjuvant, control: 150 µg per dose of injection of PBS.

As shown in Figure 4, the groups that received the antigen + adjuvant (propranolol) showed a higher survival rate compared to that of groups without the adjuvant after challenge with live and active tachyzoites. The survival rate in the group that received the recombinant SAG-1 at a concentration of 20 µg per dose of injection + propranolol is higher than that in the groups that received TLA, lower doses of r SAG-1, and in the control group. Moreover, the TLA-propranolol group of mice showed a better survival rate.

As mentioned, *T. gondii* is an intracellular parasite,^33^ and therefore, cellular responses play a more important role in comparison with humoral responses.^15^ In efforts to develop a vaccine against *T. gondii*, various antigens have been used until now, including inactivated parasites, crude or purified antigens, recombinant antigens, and those that use DNA vaccines.^34^ Moreover, various groups of adjuvants have been categorized, including carrier proteins, active immunostimulants, and vehicle adjuvants.^10^ Some adjuvants that are used in the efforts for developing *T. gondii* vaccines include alum,^35^ naltrexone,^36^ CpG,^37^ and propranolol (that when used in combination with TLA, it increased the immune responses and survival time in mice infected with *T. gondii*). There are no effective vaccines for human toxoplasmosis,^38^ and therefore, antigen and adjuvant selection has a great importance. A close relationship exists between the nervous and immune systems, and the environment that is created by the nervous mediators has a decisive role in the orientation of the immune responses to Th1 and Th2 profiles. The sympathetic nervous system has an important role in this respect. Various studies have reported that epinephrine and norepinephrine influence the cells of beta-adrenoceptors and decrease the production of inflammatory cytokines like IL-2, TNF-α and IFN-γ, and in fact, inhibit Th1 responses while stimulating the production of the cytokines that have an anti-inflammatory effects like IL-10 and TGF-β.^18^ Hence, if the sympathetic nervous system effects are suppressed by a competitive antagonist of beta-adrenoceptors such as propranolol, it will result in the development of Th1 responses that can be useful for controlling intracellular infections. In addition, immunization with the highly immunogenic SAG-1 is expected to induce protective immunity against infection by *T. gondii.*^3^ In this study, the immunogenicity effects of three different doses of rSAG-1 in combination with propranolol adjuvant or without it were evaluated and compared with the effects of TLA. Previous researches have demonstrated the positive influences of propranolol adjuvant in immunization against *T. gondii* infection^19^ and malaria.^18^ Our results showed that rSAG-1 in combination with propranolol enhanced the levels of splenic (lymphocyte proliferation and IFN-γ) and serum (IgG2a against TLA) Th1 relative reactors, and the maximum increase was observed in mice that received rSAG-1 at 20 µg per dose of injection in association with propranolol adjuvant, whereas such increased values were not observed in the groups treated with lower doses of r SAG-1 and in the adjuvant-free groups, indicating that propranolol induced effective Th1 immune responses. Also, propranolol-free mouse groups, did not show elevated levels of Th1 factors, while showed increased levels of splenic (IL-5) and serum (IgG1 against TLA) Th2 related reactors compared to propranolol-receiving groups, which is another confirmation for the effective role of propranolol in inducing Th1 related immune responses. Moreover, our results showed increased survival days after challenge with live tachyzoites in the groups that received propranolol, and this survival rate in the rSAG-1 at 20 µg per dose of injection in association with propranolol adjuvant group was obviously increased. A correlation between increased Th1 relative factors and survival rate further confirms the ability of propranolol in inducing Th1 responses against *T. gondii*. Furthermore, the increased survival time in the r SAG-1 + propranolol group at equal concentration as that of the TLA + propranolol group is another confirmation for the better immunogenic effects of r SAG-1.

## Conclusion

We recommend that in future studies similar to ours, for challenge test, low virulent strains of *T. gondii* via oral infection route can be used instead of tachyzoite injections until there is enough time for the evaluation of both acute and chronic toxoplasmosis, tissue cyst formation, survival time, and disease transmission. Also, considering the results of this study, the use of propranolol with ability at the shift the immune responses to Th1, can be evaluated for other microorganisms.

## Ethical Issues

The study was approved by Ethics Committee of Urmia University of Medical Sciences (ethical code: Ir.umsu.rec.1394.119).

## Conflict of Interest

Authors declare that they have no conflict of interest.

## Acknowledgments

This study was financially supported by Urmia University of Medical Sciences, Urmia, Iran (94-0-32-1686). The authors wish to thank Dr. Sh. Khademvatan, Mr. F. Babaei, and Ms. Sh. Khashaveh for their assistance; Dr. Naser Zia Ali for kindly offering the RH strain tachyzoites; and Urmia University of Medical Sciences for financing the project.
